# Research on the correlation between gut microbiota and brain cognitive function under chronic hypoxia at high altitude

**DOI:** 10.3389/fnins.2025.1600069

**Published:** 2025-06-19

**Authors:** Feng Zeng, Hanxue Li, Yan Ma, Shuang Ma

**Affiliations:** ^1^Department for Basic Medicine of Qinghai University, Xining, China; ^2^Research Center for High Altitude Medicine of Qinghai University, Key Laboratory of the Ministry of High Altitude Medicine, Laboratory for High Altitude Medicine of Qinghai Province, Key Laboratory of Applied Fundamentals of High Altitude Medicine (Qinghai-Utah Joint Key Laboratory of Plateau Medicine), Xining, China

**Keywords:** high altitude hypoxia, microbiota-gut-brain axis, gut microbiota, metabolome, cognitive impairment

## Abstract

**Background:**

Long-term exposure to high-altitude hypoxia can lead to cognitive impairment, yet the role of the gut microbiota in this process remains unclear. This study investigated the contribution of gut microbiota to cognitive dysfunction induced by chronic hypoxia.

**Methods:**

C57BL/6 J mice were assigned to four groups: control group (NC), control pseudo-germ-free group (CA), hypoxic group (HC), and hypoxic pseudo-germ-free group (HA). HC and HA groups were exposed to a hypobaric oxygen chamber simulating an altitude of 5,000 m (11% O₂) for 28 days. Control mice were housed Xining, 2,200 m altitude (16% O₂). All groups had free access to water; CA and HA groups received oral administration of a four-antibiotic cocktail in drinking water to deplete gut microbiota and establish pseudo-germ-free mouse models. Cognitive function was assessed by the Morris water maze, Expression levels of hippocampal BDNF, SYP, and PSD-95 were determined using Western blotting. H&E staining was used to observe morphological changes in colonic tissues. Gut microbiota composition and metabolic profiles were analyzed through 16S rRNA gene sequencing and metabolomics, respectively, followed by multi-omics correlation analyses.

**Results:**

Chronic hypoxia impaired learning and memory in mice, which was further exacerbated by gut microbiota depletion. This was evidenced by prolonged escape latency, and reduced expression of synaptic plasticity-related proteins. Although hypoxia induced colonic injury, pseudo-germ-free status did not aggravate colonic pathology. Hypoxia and microbiota depletion significantly altered gut microbial diversity, with cognitive impairment negatively correlated with *Morganella* and *Klebsiella* abundance and positively correlated with *Prevotella*, *Bifidobacterium* and *Lactobacillus*. Additionally, tryptophan metabolism and urea cycle were identified as critical pathways regulating chronic hypobaric hypoxia-induced cognitive dysfunction. S-adenosylhomocysteine and 2-isopropylmalic acid were pinpointed as potential biomarkers for hypoxia-induced cognitive impairment.

**Conclusion:**

These findings highlight the regulatory role of the gut microbiota in cognitive dysfunction under chronic hypoxic conditions and suggest potential microbiota-targeted strategies for preventing hypoxia-related brain injury.

## 1 Introduction

Low pressure and low oxygen have been regarded as the most significant feature of plateau environment and an important factor that jeopardizes health. The brain is the organ that is sensitive to oxygen and requires the most oxygen, and cerebral dysfunction is the most obvious symptom of high altitude environment (McKenna et al., 2022a; Rimoldi et al., 2016). Therefore, Individuals who first enter high-altitude environments often experience acute symptoms such as headache, dizziness, and memory decline. These symptoms typically subside as the body gradually adapts to the hypoxic conditions. However, several studies have reported that prolonged or repeated exposure to low-pressure hypoxia at altitudes above 4,000 m can impair higher brain functions—particularly learning and memory—by affecting oxygen-sensitive regions. Sustained hypoxemia may lead to irreversible structural damage in these areas and result in permanent cognitive dysfunction (Li and Wang, 2022; Liu et al., 2020; Magistretti and Allaman, 2018). Given the side effects of current neuroprotective drugs and the limited permeability of the blood–brain barrier, it is essential to investigate the mechanisms underlying hypoxia-induced brain injury from a multi-organ, systems-level perspective. Such insights could provide a theoretical foundation for developing practical and effective neuroprotection strategies (Park and Gao, 2024; Couzin-Frankel, 2023).

With the in-depth study of the plateau environment, researchers have found that the composition and activity of gut microorganisms are also altered in hypoxic environments and may lead to gastrointestinal dysfunction (Hillestad et al., 2022). Researchers compared the gut microbiota of mice at low and high altitude, and found that there was a significant difference in *β*-diversity, while there was no significant difference in an alpha diversity between the two groups (Zhang et al., 2018). Prolonged hypoxic exposure can increase gastrointestinal permeability and oxidative stress, leading to disruption of systemic homeostasis. More seriously, it can promote the translocation of gut microbiota products, activate systemic inflammatory responses, and affect the central nervous system (Loh et al., 2024).

Advances in gut microbiota research have led to the emergence of the microbiome–gut–brain axis, offering new insights into health and disease. Gut microbes influence bidirectional communication between the gastrointestinal tract and the brain via the vagus nerve, monoamines, the immune and endocrine systems, and microbial metabolites, thereby affecting neurobehavior, mood, and cognition (Dicks, 2022; Wang et al., 2023). Studies in germ-free mice have shown that commensal microbiota benefit host physiology, brain development, and cognitive function, highlighting their critical role in brain maturation (Olson et al., 2021). Disruption of the microbiota during key developmental windows impairs gut–brain communication and can result in lasting behavioral deficits (Qu et al., 2025; Seki et al., 2021; Yang et al., 2024). Additionally, germ-free models have helped uncover the role of gut microbes in establishing brain–gut connections. Emerging evidence shows that stress-induced inflammation impairs brain function and mental health, with gut microbiota acting as a mediator between stress responses, inflammation, depression, and anxiety (Luczynski et al., 2016; Roubalová et al., 2024; Roussin et al., 2024). However, the precise mechanisms by which the gut microbiota contributes to central nervous system (CNS) injury remain poorly understood. Moreover, studies exploring how the gut–brain axis operates under chronic hypoxia, especially at high altitudes, are still limited.

Therefore, in this study, we established a chronic hypoxic exposure model using C57BL/6 J mice and constructed a pseudo-germ-free mouse model by feeding them with water containing antibiotics. Through the Morris water maze test, we explored the effects of hypoxia and alterations in the gut microbiota on the learning and memory abilities of mice. Hematoxylin and eosin staining was used to investigate the effects of hypoxia and changes in the gut microbiota on the colonic tissue of mice. Western blot techniques were employed to explore the impacts of hypoxia and changes in the gut microbiota on the protein expression levels of BDNF, SYP, and PSD-95 in mouse brain tissues. Multi-omics analysis was carried out to explore the correlations among the learning and memory ability of mice, the gut microbiota, and the metabolites of the gut microbiota. This study aims to identify biomarkers of learning and memory impairment from the perspective of the gut microbiota, so as to provide new ideas for the prevention and treatment of high-altitude hypoxic brain injury.

## 2 Materials and methods

### 2.1 Animal treatments

C57BL/6J mice were randomly divided into 4 groups (*n* = 40); control group (NC), hypoxic group (HC), control pseudo aseptic group (CA), and hypoxic pseudo aseptic group (HA); mice in HC and HA groups were placed in a hypobaric chamber (DYC-300, Guizhou Feng Lei Oxygen Chamber Co., Ltd., Guizhou, China) to simulate a hypoxic environment with 5,000 m (405 mmHg, PO_2_ is 84.7 mmHg, with 11% Oxygen), and were exposed continuously for 4 weeks, the mice in NC and CA groups were housed in Xining with 2,200 m (582 mmHg, partial oxygen pressure (PO_2_) is 121.6 mmHg with 16% Oxygen), and 40 SPF-grade male C57BL/6J mice of 6 weeks of age, weighing 20 ± 2 g, were purchased from Xi’an Huishi Bio-technology Co, Ltd. [SCXK(SU)2020-0009]. and free water intake. All experimental animal management and experimental operations were in compliance with the regulations for the management of experimental animals raised at Qinghai University [SYXK(Qing)2020-0001] and approved by the Experimental Animal Ethics Committee of Plateau Medical Center.

### 2.2 Antibiotic treatment

After grouping, antibiotics were used to eliminate the intestinal microbiota of mice. The CA and HA groups were given that antibiotic (ABx) mixtures (vancomycin 0.5 g/L, ampicillin 1 g/L, neomycin 1 g/L, metronidazole 1 g/L) prepared by distilled water for 28 d, we promptly collected fecal samples from four groups for comparative analysis of gut microbiota.

### 2.3 The Morris water maze experiment

The Morris water maze apparatus consisted of a circular pool (120 cm in diameter, 50 cm in height) and a hidden circular platform (9 cm in diameter, 27 cm in height). Water temperature was maintained at 21 ± 1°C, and food-grade titanium dioxide was added to make the water opaque. The platform was submerged 1 cm below the surface to remain invisible to the mice. Visual cues of different shapes, colors, and sizes were placed on the surrounding walls to divide the pool into four quadrants, serving as spatial references. A video camera was mounted above the pool, and mouse movements were tracked using EthoVision XT software (Noldus Information Technology Co., Ltd., Beijing, China).

The experiment comprised two phases: acquisition (navigation) and probe (spatial memory) trials. During the acquisition phase, mice were trained to locate the hidden platform over five consecutive days, with four trials per day. In each trial, mice were released from one of the four quadrants, facing the wall. If a mouse located the platform within 60 s, its escape latency was recorded and it was allowed to remain on the platform for 15 s. If the platform was not found, the mouse was guided to it and also allowed to rest for 15 s to observe spatial cues. Trials were spaced 20 min apart to prevent fatigue. In the probe phase, conducted 24 h after the final training session, the platform was removed. Mice were placed into the pool from the quadrant furthest from the original platform location and allowed to swim freely for up to 120 s. The time taken to reach the former platform location was recorded. This experiment was performed during the final week of modeling.

### 2.4 Histological evaluation

The colon and brain tissues were fixed with 4% paraformaldehyde, embedded in paraffin, and sectioned at a thickness of 5 μm. The sections were stained with hematoxylin and eosin (HE), and histopathological photography was performed using a histopathological microscope (BX53, Olympus Corporation, Tokyo, Japan).

### 2.5 Western blot

Mice were anesthetized via intraperitoneal injection of 20% ursodiol at a dose of 0.5 mL/100 g. Following anesthesia, the animals were decapitated, and brain tissues were rapidly collected on ice, frozen in liquid nitrogen, and stored at −80°C. Protein was extracted from one hemisphere. Tissue was homogenized in 200–300 μL of lysis buffer for 15 s, lysed on ice for 20 min, and centrifuged at 12,000 g for 15 min at 4°C. The supernatant was collected, mixed with 5 × loading buffer, boiled at 100°C for 5 min, and stored at −20°C. Protein samples were separated by SDS-PAGE and transferred to PVDF membranes. Membranes were blocked with 5% skim milk for 1 h and incubated overnight at 4°C with the following primary antibodies: BDNF (1:1000, ab108319, Abcam), SYP (1:40000, 17,785-1-AP, Proteintech), PSD-95 (1:10000, 20,665-1-AP, Proteintech), and *β*-actin (1:10000, 20,536-1-AP, Proteintech). After five washes with 1 × TBST (5 min each), membranes were incubated with secondary antibodies at room temperature for 1 h, washed again, and visualized using ECL chemiluminescence. Finally, band intensities were quantified using ImageJ software. The relative expression level of each target protein was calculated as the ratio of the gray value of the target band to that of the internal reference band.

### 2.6 16S rRNA gene sequencing and analysis

Fecal samples were collected and stored on dry ice. Total DNA was extracted using the OMEGA Soil DNA Kit (M5635-02) with a final elution volume of 100 μL. DNA quality and integrity were assessed using a NanoDrop NC2000 spectrophotometer and 1% agarose gel electrophoresis. The V3–V4 region of the 16S rRNA gene was amplified using primers 338F and 806R. Amplicons were quantified with the Quant-iT PicoGreen dsDNA Assay Kit and purified using BECKMAN AMPure XP beads. Sequencing was performed on the Illumina MiSeq platform (2 × 250 bp). Alpha diversity indices (Chao1, Observed species, Shannon, Simpson, Faith’s PD, Pielou’s evenness, and Good’s coverage) were calculated based on ASVs using QIIME2, and visualized with box plots in R. Beta diversity was evaluated using UniFrac distances in QIIME2 and R. Taxonomic composition at six levels (phylum to species) was assigned with QIIME2 and displayed using bar plots. Group differences were assessed with PERMANOVA (Adonis), and LEfSe was used to identify differential taxa. Multi-omics correlation analysis was conducted using redundancy analysis (RDA), Spearman correlation, and SparCC. Correlations with |r| > 0.4 and FDR-adjusted *p* < 0.05 were considered significant. Multiple testing correction was applied using the Benjamini–Hochberg false discovery rate method.

### 2.7 Metabolomics analysis

Mouse feces were first pre-treated to extract the proteins, and then chromatography-mass spectrometry analysis was provided by Bay Spectrum Biotechnology (Shanghai, China). Namely, this included a Shimadzu Nexera X2 LC-30 AD system equipped with an ACQUITY UPLC HSS T3 column (1.8 μm, 2.1 × 50 mm column, Waters) and a triple quadrupole mass spectrometer (5,500 QTRAP, AB SCIEX). Metabolites were detected in electrospray negative and positive ionization modes. The mass spectrometry conditions were set as follows: negative ionization: source temperature 550°C, ion source Gas1 (GAS1): 40, ion source Gas2 (GAS2): 50, Curtain gas (CUR): 35, Ion Spray Floating Voltage (ISVF): −4,500 V; positive ionization: source temperature 550°C, ion source Gas1 (GAS1): 40, and Ion Source Gas2 (GAS2): 50, Curtain Gas (CUR): 35, Ion Spray Floating Voltage (ISVF): 5500 V. Conversions were detected using MRM mode. Raw MRM data of MT1000 KIT metabolites were extracted using Multi Quant 3.0.2 software to obtain the peak area of each metabolite.

### 2.8 Statistical methods

In this experiment, all data were analyzed and plotted using Graphpad Prism 9.3 software. The data were expressed as mean ± standard deviation ( $\bar{X}$ ± s). The normality distribution was tested by the Shapiro–Wilk test. For the comparison between two groups, the t-test or Mann–Whitney U test was applied. *p < 0.05 indicated that the difference was statistically significant. In the analysis of omics data, the Wilcoxon rank-sum test was employed. Statistical significance was defined as a false discovery rate (FDR)-adjusted *p*-value < 0.05. For correlation analysis, the Spearman rank correlation coefficient was used.

## 3 Results

### 3.1 Changes of behavioral tests and learning and memory-related proteins in mice

#### 3.1.1 Behavioral tests

**Figure 1:**
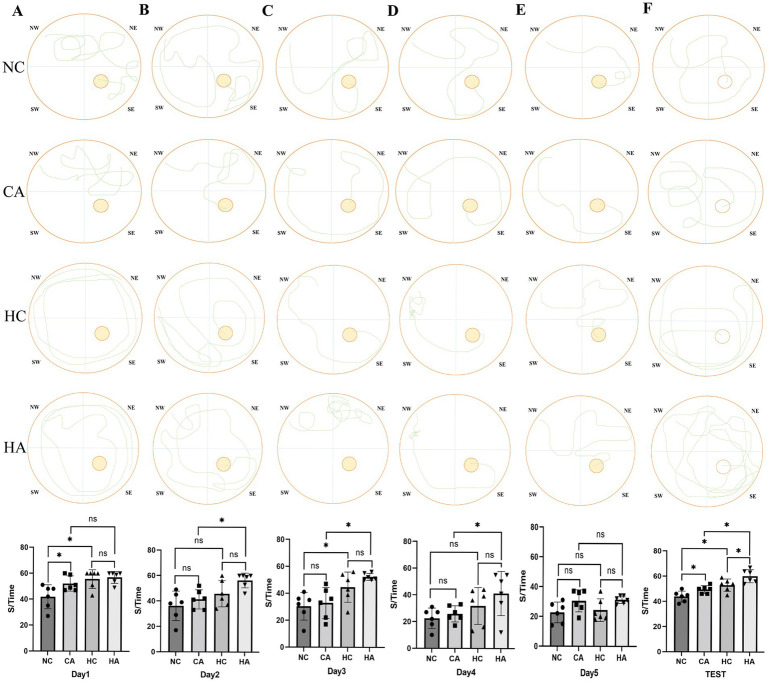
Changes in escape latency of the Morris water maze test in mice. **(A)** Escape latency of mice in localization navigation experiment on day 1; **(B)** Escape latency of mice in localization navigation experiment on day 2; **(C)** Escape latency of mice in localization navigation experiment on day 3; **(D)** Escape latency of mice in localization navigation experiment on day 4; **(E)** Escape latency of mice in localization navigation experiment on day 5; **(F)** Escape latency of mice in Spatial search experiment. ns: no significant difference; *p < 0.05 indicates significant difference. NC, control group; CA, control pseudo-germ-free group; HC, hypoxic group; HA, hypoxic pseudo-germ-free group.

The results from the place navigation test showed that escape latency decreased across training days in all groups (Figures 1A–D). During the first 4 days, mice in the HC and CA groups exhibited significantly longer escape latencies compared to the NC group (*p* < 0.05), and the HA group showed a longer latency than the CA group (*p* < 0.05). Although the HA group also showed a longer latency than the HC group, the difference was not statistically significant (*p* > 0.05). By day 5 (Figure 1E), no significant differences in escape latency were observed among the four groups (*p* > 0.05), suggesting that training led to gradual improvements in spatial learning. In contrast, the spatial probe test revealed significant differences among all groups (Figure 1F, *p* < 0.05), indicating persistent impairments in memory retention. These findings suggest that both chronic hypoxia and antibiotic treatment impair learning and memory in mice, and their combination may further exacerbate cognitive deficits.

#### 3.1.2 Learning and memory-related proteins

**Figure 2:**
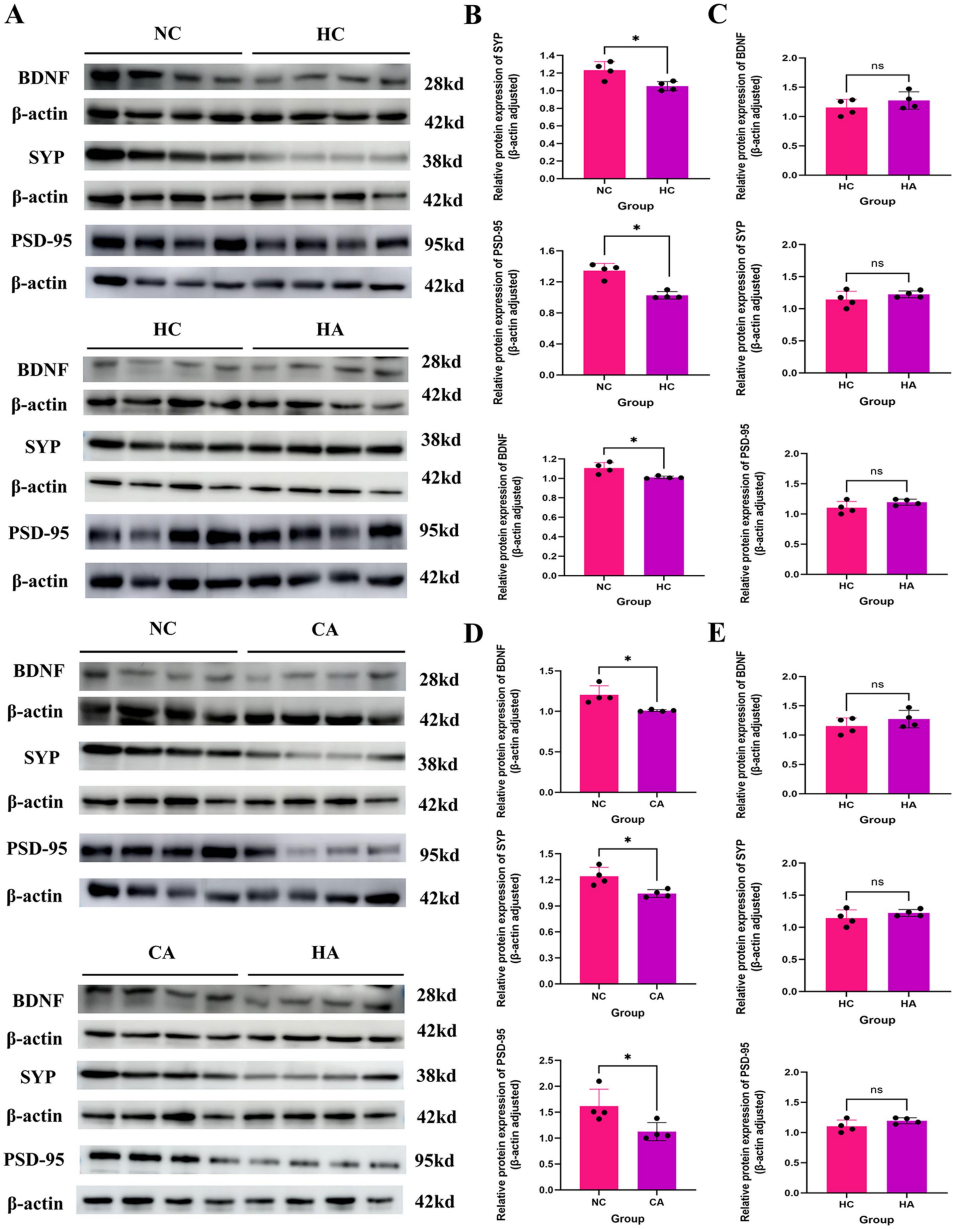
Expression of learning and memory-related proteins in mice. **(A)** WB results of BDNF, SYP, and PSD-95 protein expression; **(B)** Expression levels of BDNF, SYP, and PSD-95 proteins in NC and HC groups; **(C)** Expression levels of BDNF, SYP, and PSD-95 proteins in HC and HA groups; **(D)** Expression levels of BDNF, SYP, and PSD-95 proteins in NC and CA groups; **(E)** Expression levels of BDNF, SYP, and PSD-95 proteins in CA and HA groups. ns: no significant difference; *p < 0.05 indicates significant difference. NC: control group; CA: control pseudo-germ-free group; HC: hypoxic group; HA: hypoxic pseudo-germ-free group.

The changes in learning and memory-related proteins in mice were demonstrated via Western blot. Figures 2A,B showed that compared with the NC group, the expression levels of learning and memory-related proteins BDNF, PSD-95, and SYP in the HC group were decreased (*p* < 0.05); compared with the HC group, There was no change in the expression levels of BDNF, PSD-95, and SYP in the HA group (Figures 2A,C; *p* < 0.05); compared with the NC group, the CA group showed statistically significant decreases in the expression levels of BDNF, PSD-95, and SYP (Figures 2A,D; *p* < 0.05); compared with the CA group, the HA group had significantly reduced expression levels of BDNF, PSD-95, and SYP (Figures 2A,E; *p* < 0.05). This indicated that both chronic hypoxia and antibiotic treatment could lead to impairments in learning and memory abilities in mice.

### 3.2 Alterations in colonic tissue and gut microbiota of mice

#### 3.2.1 Mouse colon tissue

**Figure 3:**
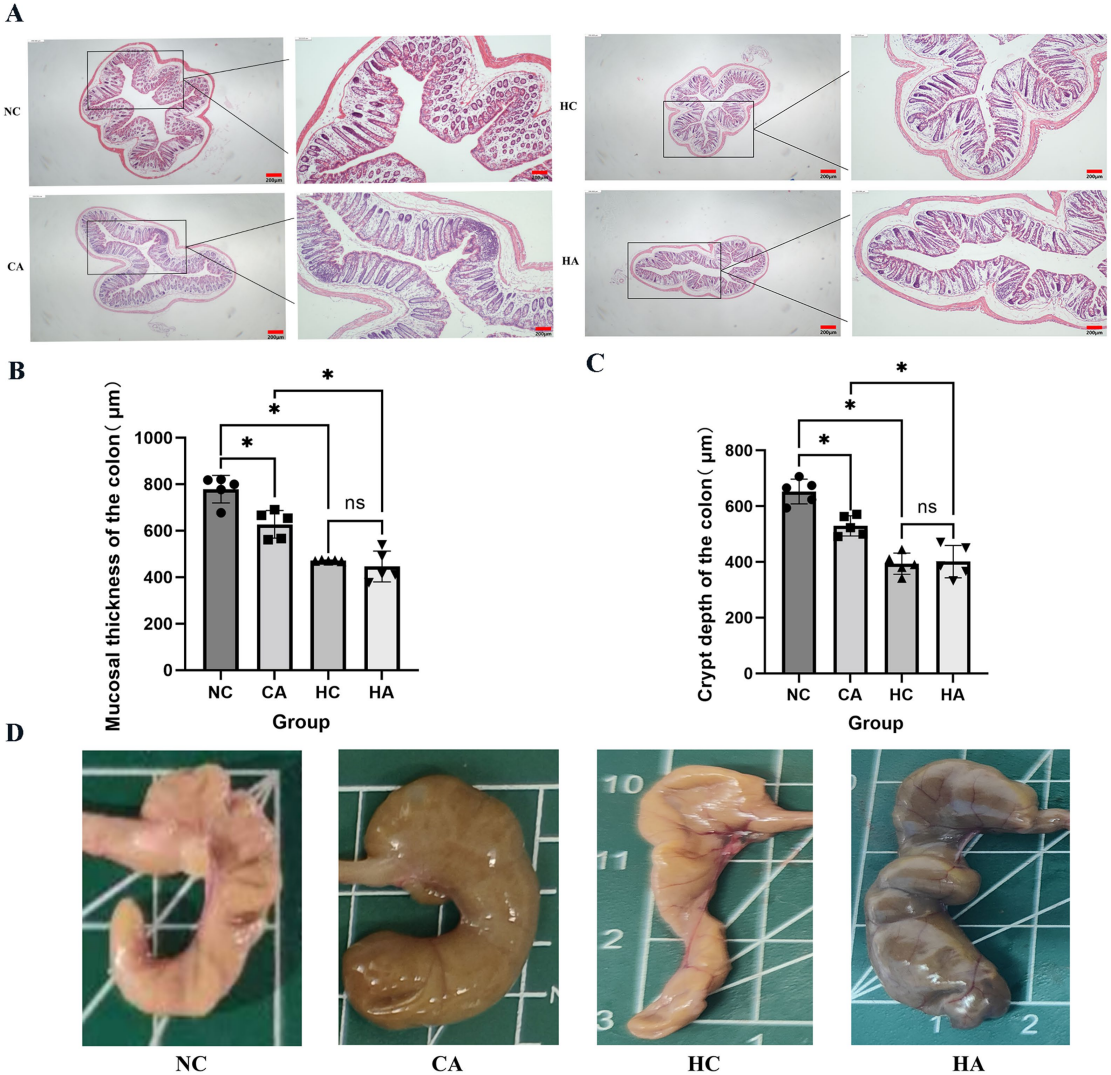
Alterations in mouse colonic tissues. **(A)** H&E staining of colonic tissues (40X; 100X); **(B)** Colonic mucosal thickness in four groups; **(C)** Colonic crypt depth in four groups; **(D)** Mouse ileocecal region. ns: no significant difference; *p < 0.05 indicates significant difference. NC, control group; CA, control pseudo-germ-free group; HC, hypoxic group; HA, hypoxic pseudo-germ-free group.

Figure 3A showed the H&E staining results of mouse colonic tissues. Compared with the NC group, the HC and CA groups exhibited Crypt architectural disruption, intestinal epithelial cell damage, and decreased mucosal thickness and crypt depth (Figures 3B,C, *p* < 0.05). When comparing the HA group with the CA group, the HA group showed further exacerbated intestinal epithelial cell damage, and more significant decreases in mucosal thickness and crypt depth. Additionally, no further damage was observed in the colon of the HA group compared with the HC group. Figure 3D showed that the ileocecal regions of the NC and HC groups had intact mucosal folds and normal coloration, while the CA and HA groups exhibited mucinous edema— a characteristic indicator of successful pseudo-germ-free model induction. These findings suggest that both chronic hypoxia and antibiotic treatment can induce colonic injury. However, antibiotic treatment under hypoxic conditions did not further aggravate the pathological damage.

#### 3.2.2 Gut microbiota

Supplementary Figure S1 demonstrates the successful depletion of intestinal microbiota following antibiotic treatment. Panels S1A–E show gel electrophoresis, bacterial DNA quantification, and reductions in ASV/OTU counts at the order, family, and genus levels, confirming the establishment of a pseudo-germ-free mouse model.

**Figure 4:**
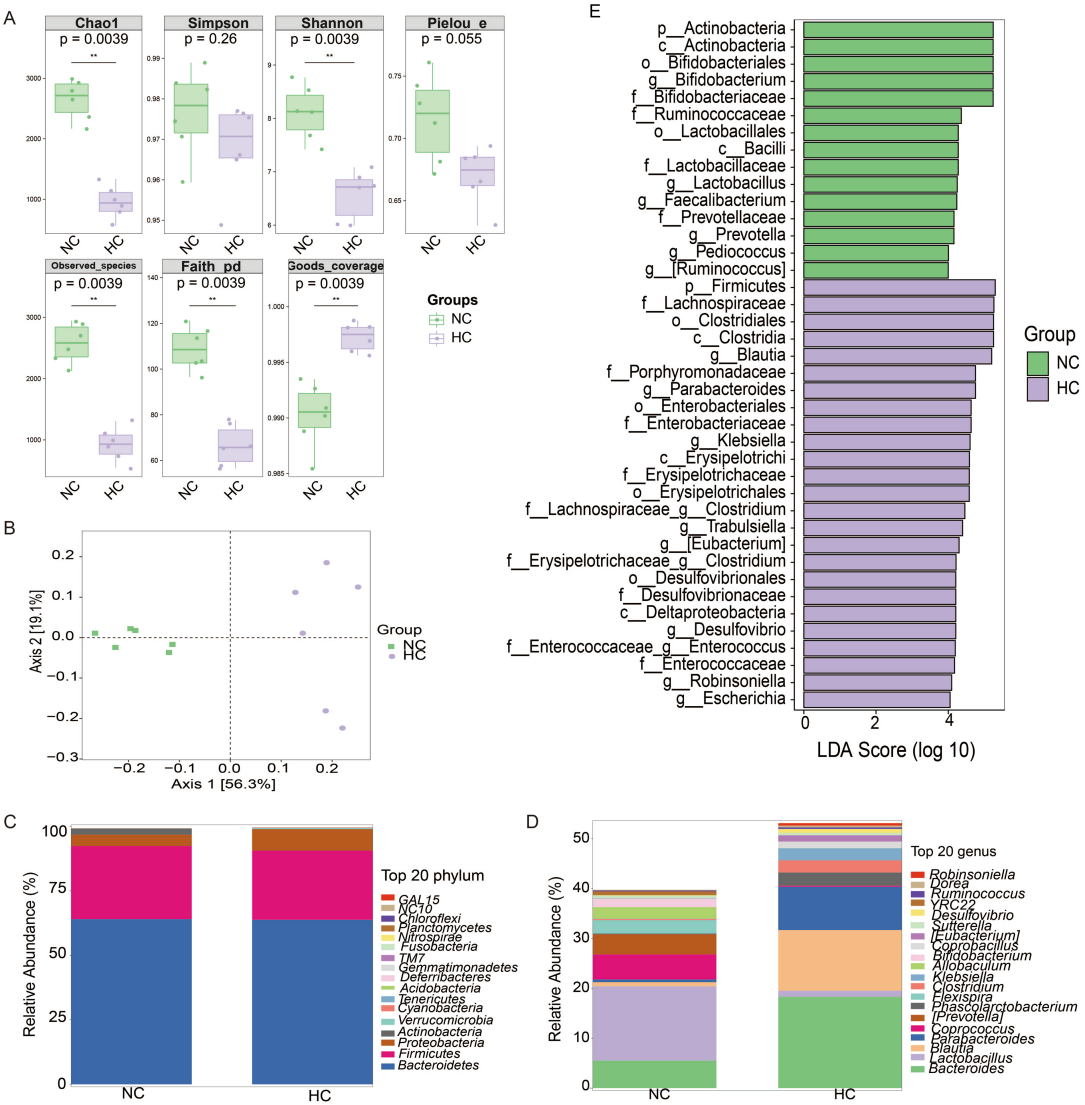
Changes in gut microbiota of mice. **(A)** Alpha diversity analysis of gut microbial communities. **(B)** Principal coordinate analysis (PCoA) of unweighted UniFrac intergroup distances. **(C)** Histogram of abundance at the phylum level of the mouse microbial community. **(D)** Histogram of abundance at the genus level of the mouse microbial community. **(E)** LEfSe differential analysis of gut microbiota at the genus level between NC and HC groups. ns: no significant difference; *p < 0.05 indicates significant difference. NC, control group; CA, control pseudo-germ-free group; HC, hypoxic group; HA, hypoxic pseudo-germ-free group.

Alpha diversity analysis revealed significant differences in microbial richness and diversity between NC vs. HC, NC vs. CA, and HC vs. HA groups (Supplementary Figures S2A, S3A, Figure 4A; *p* < 0.05). Beta diversity assessed by principal coordinate analysis (PCoA) based on unweighted UniFrac distances showed clear clustering and distinct microbial community compositions among the same group comparisons (Supplementary Figures S2B, S3B, Figure 4B). Taxonomic profiling at the phylum level revealed that *Bacteroidetes* dominated the NC and HC groups (mean relative abundances: 64.06 and 63.80%, respectively), while *Proteobacteria* were predominant in the CA and HA groups (98.89 and 95.66%) (Supplementary Figures S2C, S3C, Figure 4C). At the genus level (Figure 4D), S24-7 and *Lactobacillus* were most abundant in the NC group (51.40, 14.90%), whereas S24-7, *Bacteroides*, and *Blautia* were predominant in the HC group (35.24, 18.32, 12.16%). In contrast, the CA and HA groups exhibited markedly reduced genus-level diversity, with most taxa belonging to *Proteobacteria* (Supplementary Figures S2D, S3D, Figure 4D).

LEFSe differential analysis identified 16 differentially abundant genera. The NC group had increased abundances of *Bifidobacterium*, *Lactobacillus*, *Faecailbacterium*, *Prevotella*, *Pediococcus*, and *Ruminococcus*, while the HC group showed enrichment of *Blautia*, *Parabacteriales*, *Klebsiella*, *Clostridium*, *Trabulsiella*, *Eubacterium*, *Desulfovibrio*, *Enterococcus*, Robinsoniella, and *Escherichia* (Figure 4E).

#### 3.2.3 Gut microbiota metabolites

**Figure 5:**
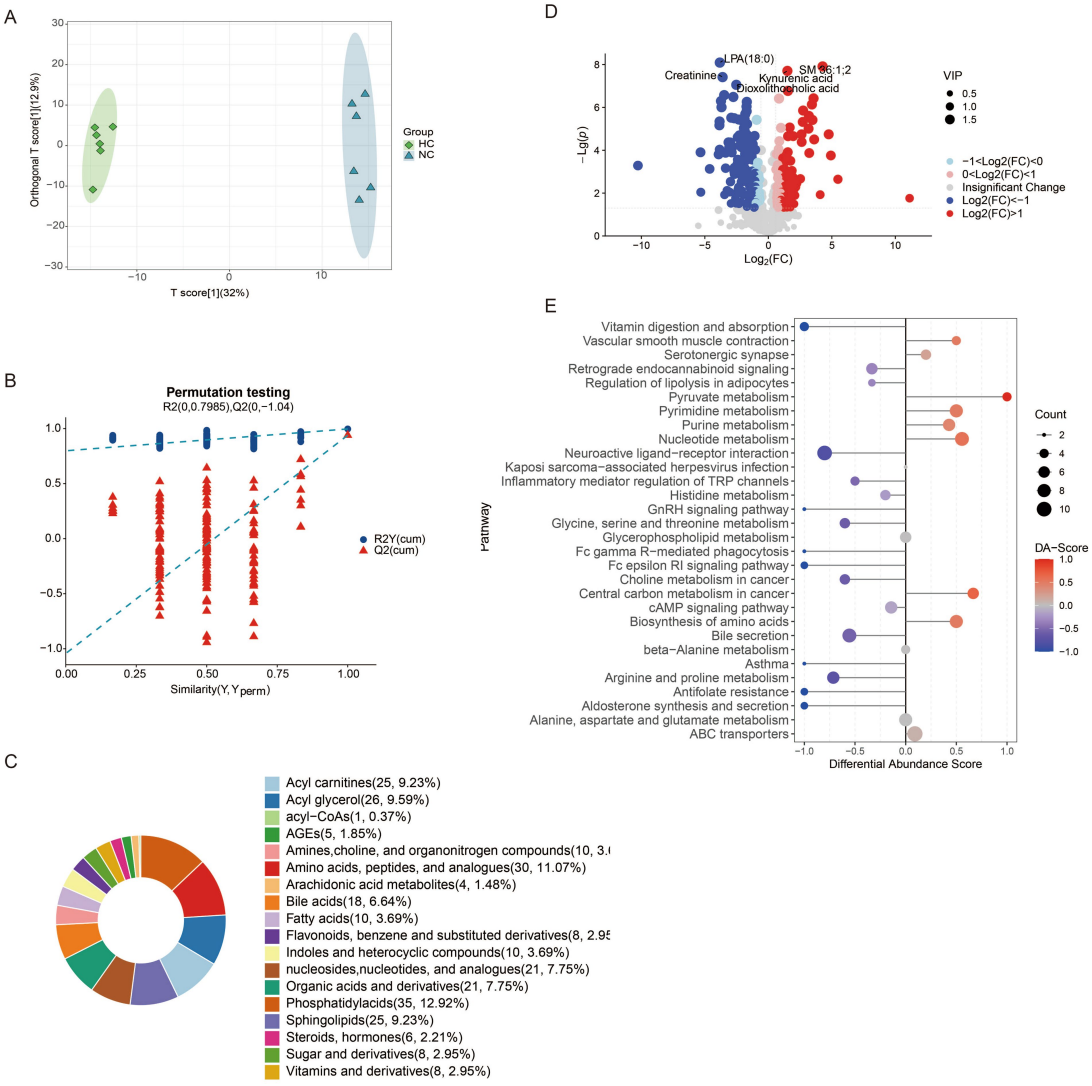
Changes of metabolites of gut microbiota in mice. **(A)** Distinction of OPLS-DA model based on the metabolic profiles of gut microbiota (*n* = 6); **(B)** Robustness of the OPLS-DA model was assessed by using a randomized permutation test with 200 trials; **(C)** Differential metabolite classification; **(D)** Heat maps of the different metabolites in the feces of mice; **(E)** Differential scoring of KEGG pathway abundance. ns: no significant difference; *p < 0.05 indicates significant difference. NC, control group; CA, control pseudo-germ-free group; HC, hypoxic group; HA, hypoxic pseudo-germ-free group.

Metabolomics analysis revealed distinct metabolic profiles among the four mouse groups, with orthogonal partial least squares discriminant analysis (OPLS-DA) showing clear separation between all pairwise comparisons (Supplementary Figures S4A, S5A, Figure 5A). A 200-permutation randomization test confirmed the robustness of the model and excluded overfitting (Supplementary Figures S4B, S5B, Figure 5B). In the comparison between NC and HC groups, 271 differential metabolites were identified, with 157 downregulated and 114 upregulated (Figure 5C). These metabolites were primarily categorized into phosphatidylacids, amino acids, acyl glycerols, acyl carnitines, and sphingolipids (Figure 5D). Pathway analysis revealed upregulated pathways, including pyruvate metabolism, pyrimidine metabolism, nucleotide metabolism, and amino acid biosynthesis, while downregulated pathways included neuroactive ligand-receptor interaction, inflammatory mediator regulation of TRP channels, and glycine metabolism (Figure 5E). A comparison of NC and CA groups identified 310 differential metabolites, with 113 downregulated and 197 upregulated (Supplementary Figure S4C), primarily categorized as amino acids, bile acids, acyl carnitines, and phosphatidylglycerols (Supplementary Figure S4D). Upregulated pathways included nucleotide metabolism, ABC transporters, purine metabolism, and cysteine/methionine metabolism, while downregulated pathways involved glycine/serine/threonine metabolism and estrogen signaling (Supplementary Figure S4E). In the HC vs. HA comparison, 299 differential metabolites were detected, with 62 downregulated and 237 upregulated (Supplementary Figure S5C), primarily classified as amino acids, peptides, organic acids, nucleosides, and bile acids (Supplementary Figure S5D). Upregulated pathways included nucleotide metabolism, amino acid biosynthesis, and neuroactive ligand-receptor interaction, while downregulated pathways included glucagon signaling and glycolysis (Supplementary Figure S5E).

### 3.3 Correlation analysis between gut microbiota, fecal metabolites and brain behavioral data

Supplementary Figures S6A–C found that the escape latency of mice in the NC group compared to the HC group was significantly correlated with genera such as *Klebsiella*, *Morganella*, and *Parabacteroides*; and negatively with *Prevotella*, *Bifidobacterium* and *Lactobacillus*; A total of 41 metabolites were closely related to the escape latency of mice. Metabolites such as *γ*-aminobutyric acid (GABA) and Tryptamine were positively correlated with the escape latency of mice; while metabolites such as 2-Isopropylmalic acid, S-Adenosylhomocysteine, Xanthurenic acid, and Succinic acid were negatively correlated with the escape latency of mice. Supplementary Figures S7A–C shows that the escape latency of mice in the NC group compared with the CA group was negatively correlated with the escape latency of *Flexispira*, *Adlercreutzia*, *Bifidobacterium* and *Prevotella*; a total of 59 metabolites were closely related to the escape latency of mice, Metabolites such as S-Adenosylhomocysteine and 2-Isopropylmalic acid were negatively correlated with the escape latency of mice. Supplementary Figures S8A–C found that the escape latency of mice in the HC group compared with the HA group was similar to that of *Enterobacter*, *Morganella*, *Shigella*; negative correlation with *Akkermansia*, *Clostridium*, and *Coprobacillus*; 21 metabolites were closely related to the escape latency of mice, Metabolites such as 2-Isopropylmalic acid and Cytidine monophosphate (CMP) were negatively correlated with the escape latency of mice (Supplementary Figures S6–S8).

## 4 Discussion

Cerebral dysfunction is one of the most prominent symptoms observed in high-altitude environments (McKenna et al., 2022b). Prolonged or repeated exposure to hypoxia at elevations above 4,000 m for over 1 month can cause irreversible pathological damage to brain tissues, ultimately resulting in permanent cognitive impairment (Liu et al., 2020; Magistretti and Allaman, 2018). In the present study, we observed that in the Morris water maze experiment, the escape latency of mice in HC group was significantly longer than that of mice in NC group, with a statistically significant difference, suggesting that a chronic hypoxic environment can result in a decline in the learning and memory abilities of mice (Zhao et al., 2022). Similarly, the CA group also exhibited prolonged escape latency compared to the NC group, suggesting that gut microbiota depletion via antibiotic treatment alone can impair cognitive function (Fishbein et al., 2023). Notably, the HA group exhibited a further increase in escape latency compared to the HC group, consistent with the findings of Zhang et al. (2023), implying that combined chronic hypoxia and antibiotic treatment exacerbates cognitive impairment. Although a pseudo-germ-free model was established, this outcome indicates a synergistic detrimental effect. Although in our study, a pseudo-germ-free mouse model was established. This phenomenon implies that the combined treatment of chronic hypoxia and antibiotics exacerbates the impairment of learning and memory abilities in mice. However, some studies have indicated that in mice treated with broad-spectrum antibiotics, the cognitive impairment was alleviated under the condition of short-term (5 days) acute hypoxia (Olson et al., 2021). This discrepancy may stem from differences in hypoxia duration. Acute hypoxia is a short-term process, and antibiotic interference may protect the learning and memory of mice by inhibiting the endotoxemia caused by the excessive proliferation of gut microbiota. Conversely, chronic hypoxia is a long-term process, and antibiotic treatment can lead to a remarkable reduction in the diversity of gut microbiota, which further impairs the learning and memory abilities of mice. To explore the molecular basis of these findings, we assessed the expression of learning-and memory-related proteins (BDNF, SYP, and PSD-95). The NC group showed significantly higher expression levels than both the HC and CA groups, further supporting the notion that chronic hypoxia and gut microbiota depletion independently impair cognitive function (Fröhlich et al., 2016). However, no significant differences were observed between the HC and HA groups, which may be related to the difference in the part from which the material was taken.

Chronic hypoxia has been shown to alter the composition and activity of the gut microbiota and may contribute to gastrointestinal dysfunction (Qaid et al., 2017; Sorboni et al., 2022). In our study, H&E staining revealed crypt architectural disruption, damaged intestinal epithelial cells, and decreased mucosal thickness and crypt depth in both CA and HC groups compared with NC mice. However, no additional damage was observed in the HA group compared with the HC group, suggesting that antibiotic treatment under hypoxic conditions does not further exacerbate colonic injury. This observation is consistent with Zhang et al. (2023). It indicates that under chronic hypoxia, the integrity of the epithelial layer of the colon tissue in mice will be impaired, and the depletion of gut microbiota after antibiotic treatment will also damage the colon tissue. However, the combined treatment of chronic hypoxia and antibiotics will not further exacerbate the pathological manifestations. Studies have revealed that interfering with gut microbiota during the critical growth period of model animals can disrupt the normal communication between the gut and the brain, and may have long-term negative impacts on brain behavior (Qu et al., 2025; Seki et al., 2021; Yang et al., 2024). In our analysis, significant differences in *α*-and *β*-diversity were observed among the groups, consistent with prior reports. However, Zhang et al. (2018) Found that there was a significant difference in β-diversity between low altitude and high altitude groups, while there was no significant difference in α-diversity. Meanwhile, hypoxic exposure can lead to significant changes in the structure of gut microbiota in mice. In particular, the decline in learning and memory caused by exposure to a chronic hypoxic environment is related to the imbalance of the gut microbiota (Olson et al., 2021). In this study, Taxonomic analysis showed that *Bacteroidetes* dominated the NC and HC groups (64.06 and 63.80%, respectively), whereas *Proteobacteria* predominated in the CA and HA groups (98.89 and 95.66%). These microbial changes were accompanied by metabolomic alterations. OPLS-DA score plots and permutation tests revealed distinct clustering among NC vs. HC, NC vs. CA, and HC vs. HA groups, confirming the presence of differential metabolites without model overfitting. Combining the results of the above behavioral experiments, it is shown that chronic hypoxia not only impairs cognitive function but also affects the composition of gut microbiota and intestinal metabolites. Antibiotic treatment can also impair cognitive function and disrupt the composition of gut microbiota and intestinal metabolites.

Additionally, in this study, we also found that the learning and memory ability of mice was negatively correlated with *Morganella*, *Klebsiella*, etc., and positively correlated with *Prevotella*, *Bifidobacterium*, and *Lactobacillus*. *Morganella* is a conditional pathogen, and LPS secreted by it can trigger a strong immune response in the blood of patients with depression. This inflammatory response may indirectly have a negative impact on cognitive function by affecting the metabolism of neurotransmitters, neural plasticity, and the neuroendocrine system, etc. (Chang et al., 2024). Some studies have shown that *Klebsiella* can affect the production of serum ferritin in nerve cells and act on glial cells, leading to cognitive impairment (Rosell-Díaz et al., 2023). *Bifidobacterium* can produce a variety of tryptophan metabolites, participate in tryptophan metabolism, and affect cognitive function. Studies have indicated that increasing the intake of *Bifidobacterium* in the group with cognitive abnormalities can improve cognitive dysfunction (Shi et al., 2022). *Lactobacillus* participates in the tryptophan metabolic pathway by affecting the changes in the concentration of kynurenine, leading to cognitive impairment (Rudzki et al., 2019). Some studies have also shown that supplementing with probiotics rich in *Lactobacillus* can alleviate cognitive dysfunction (Mosquera et al., 2024). *Prevotella* is also a conditional pathogen, and existing studies support its importance in metabolic reactions, yet the research on the specific mechanism remains unclear (Tett et al., 2021). In addition to these identified bacteria, there are still many unknown species that have not been characterized, and further research is needed to elucidate their roles.

Amino acids, as fundamental biological macromolecules in the living system, are not only the core components of the cell structure, but also regulate the growth, development, and repair processes of organisms through their complex metabolic networks (Chang et al., 2024). The differential metabolomics analysis in this study showed that the abundance changes of amino acid metabolites such as Carbamoyl phosphate, Cinnamoyl glycine, S-Adenosylhomocysteine, and amino acid derivatives were significantly positively correlated with the cognitive function of mice. It is worth noting that the abnormal accumulation of these metabolites indicates an enhancement of amino acid catabolism, which may reflect an increased demand for specific amino acids in the body (Ajaz et al., 2021). However, when exposed to a chronic hypoxic environment, gut microbiota can cause amino acid metabolic disorders, leading to the imbalance of the host’s intestinal homeostasis through increasing intestinal permeability and inflammatory responses (Gong et al., 2020; Li et al., 2020). S-Adenosylhomocysteine is the product of the methylation reaction in which S-adenosylmethionine acts as a methyl donor, including the methylation of DNA, histones, and other proteins (Caudill et al., 2001). Both *in vivo* and *in vitro* indicates that an increase in the level of S-Adenosylhomocysteine can cause endothelial cell dysfunction (Castro et al., 2005). However, some studies have shown that a decrease in the level of S-Adenosylhomocysteine in patients with Alzheimer’s disease severely impairs the metabolism and brain function of the patients (Morrison et al., 1996). Carbamoyl phosphate is a precursor for the synthesis of pyrimidines and arginine. Abnormal pyrimidine metabolism leads to insufficient nucleotide synthesis, which may affect the normal function of neurons and thus impact cognitive ability (Charlier et al., 2018). A decrease in arginine synthesis results in a reduction in urea production, causing disorders in the body’s nitrogen and ammonia balance and leading to urea cycle disorders, thereby impairing cognitive function (Enns, 2008). 2-Isopropylmalic acid is a key intermediate product in the degradation of leucine, indirectly participating in the energy metabolic pathway and capable of improving the growth of animals (Huang et al., 2023). In this study, the body weight of mice in the hypoxia and antibiotic treatment groups decreased, indicating that 2-Isopropylmalic acid may affect cognitive dysfunction by influencing the growth and development of animals.

Furthermore, we also found that Succinic acid was positively correlated with the cognitive function of mice, suggesting that under hypoxic conditions, the tricarboxylic acid cycle is affected, resulting in a decrease in ATP production, affecting the energy metabolism of cells, and leading to cognitive impairment (Ma et al., 2023). Studies have found that tryptophan metabolites can bind to Aryl Hydrocarbon Receptor (AhR), activate the immune system, exert antioxidant and anti-inflammatory effects, and may regulate gut microbiota and metabolome (Sorboni et al., 2022). In this study, the cognitive function of mice was positively correlated with Xanthurenic acid and negatively correlated with Tryptamine. Xanthurenic acid is formed from 3-hydroxykynurenine in the metabolism of tryptophan. It can reduce the release of glutamate by acting on specific G protein-coupled receptors and affecting the intracellular Ca2 + concentration, thus causing cognitive impairment (Sathyasaikumar et al., 2017). Tryptamine is an amine formed by the decarboxylation of tryptophan. Hypoxia can increase the production of tryptamine, thereby inhibiting the decrease in the metabolites of the kynurenine pathway, such as kynurenine, 3-hydroxykynurenine, and their decomposition products (Mohapatra et al., 2021). The experimental results in this study are consistent with this. After the mice were treated with antibiotics, under the control condition, the amino acid metabolic pathway played a relatively important role in cognitive impairment, possibly due to the depletion of gut microbiota leading to amino acid metabolic disorders, increased intestinal permeability, and inflammatory responses, which in turn caused cognitive dysfunction. Under hypoxic conditions, the carbohydrate metabolism played a relatively important role in cognitive impairment. Hypoxia itself can lead to carbohydrate metabolic disorders, causing the accumulation of lactic acid, leading to a series of metabolic disorders, and activating pathways such as Hypoxia-Inducible Factor 1 Alpha (HIF-1α), Hypoxia-Inducible Factor 2 Alpha (HIF-2α), and AMPK, thus further impairing cognitive function (Gong et al., 2020). However, the number of literatures on the impact of changes in the intestinal environment on the physiological metabolism of the host is limited. This speculation requires a large number of experimental verifications, and further experimental verifications are also needed for the study of the specific mechanisms.

This study also has several undeniable limitations. First, although we observed reduced expression of key synaptic plasticity–related proteins such as BDNF, SYP, and PSD-95, we did not further assess their classical downstream signaling pathways (e.g., PI3K/Akt and MAPK/ERK), limiting our understanding of the underlying molecular mechanisms. Second, we used an antibiotic-based pseudo-germ-free mouse model to deplete gut microbiota. Although effective, this approach cannot fully replicate the germ-free state and may introduce off-target effects due to antibiotic exposure. The use of germ-free mice or fecal microbiota transplantation (FMT) models in future research would enhance the causal inference regarding the role of gut microbiota. Third, the duration of chronic hypoxia exposure in this study was relatively short (28 days), which may not have been sufficient to induce observable morphological damage in hippocampal neurons. Although cognitive impairments were evident in behavioral testing (e.g., Morris water maze), structural alterations in the hippocampus may require longer hypoxic exposure to manifest. Future studies with prolonged exposure periods (e.g., 6 months or more) are warranted to determine the critical time window for hippocampal injury and to investigate whether neuronal damage worsens over time.

## 5 Conclusion

These results provide evidence for the connection among the microbiome, the gut-brain axis, and the hypoxia caused by the high-altitude environment. By constructing a pseudo-germ-free mouse model, it was found that *Morganella* and *Klebsiella* are negatively correlated with brain cognitive function. Under the condition of abnormal proliferation, they will lead to damage to brain cognitive function. *Bifidobacterium*, *Prevotella*, and *Lactobacillus* are positively correlated with brain cognitive function and may play a protective role in brain cognitive function. In addition, tryptophan metabolism, urea cycle pathway, etc., may be important pathways for regulating the cognitive dysfunction induced by chronic hypoxia at high altitudes. S-Adenosylhomocysteine and 2-Isopropylmalic acid may be potential biomarkers in the cognitive dysfunction induced by chronic hypoxia. These findings reveal the regulatory role of gut microbiota in cognitive dysfunction under chronic hypoxic conditions, and provide potential gut microbiota and metabolic pathway-targeted intervention strategies for the prevention of hypoxia-related brain injury.

## Data Availability

The datasets presented in this study can be found in online repositories. The names of the repository/repositories and accession number(s) can be found in the article/Supplementary material.

## Supplementary material

SUPPLEMENTARY FIGURE S1 Quantitative verification of pseudo-germ-free mice. **(A)** The results of the bacterial DNA gel electrophoresis; **(B)** The number of ASVs/OTUs in four groups; **(C)** The results of bacterial DNA content; **(D)** The number of order in four groups; **(E)** The number of family in four groups; **(F)** The number of genus in four groups. ns, no significant difference; **p* < 0.05 indicates significant difference. NC, control group; CA, control pseudo-germ-free group; HC, hypoxic group; HA, hypoxic pseudo-germ-free group.

SUPPLEMENTARY FIGURE S2 Changes in gut microbiota of mice. **(A)** Alpha diversity analysis of gut microbial communities; **(B)** Principal coordinate analysis (PCoA) of unweighted UniFrac intergroup distances; **(C)** histogram of abundance at the phylum level of the mouse microbial community. **(D)** histogram of abundance at the genus level of the mouse microbial community. ns, no significant difference; **p* < 0.05 indicates significant difference. NC, control group; CA, control pseudo-germ-free group; HC, hypoxic group; HA, hypoxic pseudo-germ-free group.

SUPPLEMENTARY FIGURE S3 Changes in gut microbiota of mice. **(A)** Alpha diversity analysis of gut microbial communities; **(B)** Principal coordinate analysis (PCoA) of unweighted UniFrac intergroup distances; **(C)** histogram of abundance at the phylum level of the mouse microbial community; **(D)** histogram of abundance at the genus level of the mouse microbial community. ns, no significant difference; **p* < 0.05 indicates significant difference. NC, control group; CA, control pseudo-germ-free group; HC, hypoxic group; HA, hypoxic pseudo-germ-free group.

SUPPLEMENTARY FIGURE S4 Changes of metabolites of gut microbiota in mice. **(A)** Distinction of OPLS-DA model based on the metabolic profiles of gut microbiota (*n* = 6); **(B)** Robustness of the OPLS-DA model was assessed by using a randomized permutation test with 200 trials; **(C)** Differential metabolite classification; **(D)** Heat maps of the different metabolites in the feces of mice; **(E)** Differential scoring of KEGG pathway abundance. ns, no significant difference; **p* < 0.05 indicates significant difference. NC, control group; CA, control pseudo-germ-free group; HC, hypoxic group; HA, hypoxic pseudo-germ-free group.

SUPPLEMENTARY FIGURE S5 Changes of metabolites of gut microbiota in mice. **(A)** Distinction of OPLS-DA model based on the metabolic profiles of gut microbiota (*n* = 6); **(B)** Robustness of the OPLS-DA model was assessed by using a randomized permutation test with 200 trials; **(C)** Differential metabolite classification; **(D)** Heat maps of the different metabolites in the feces of mice; **(E)** Differential scoring of KEGG pathway abundance. ns, no significant difference; **p* < 0.05 indicates significant difference. NC, control group; CA, control pseudo-germ-free group; HC, hypoxic group; HA, hypoxic pseudo-germ-free group.

SUPPLEMENTARY FIGURE S6 Correlation analysis between gut microbiota, fecal metabolites and brain behavioral data. **(A)** Heat map of correlation analysis between latent escape period and gut microbiota in mice (NC vs HC); **(B)** Heat map of correlation analysis between latent escape period and intestinal metabolites in mice (NC vs HC); **(C)** differential flora and differential metabolites correlation analysis heat map (NC vs HC). (The correlation coefficients *R* are shown in color, where *R* > 0 indicates positive correlation, which is shown in brown or yellow or red, and *R* < 0 indicates negative correlation, which is shown in dark blue or brown or azure; the darker the color, the stronger the correlation). ns, no significant difference; **p* < 0.05 indicates significant difference. NC: control group; CA: control pseudo-germ-free group; HC: hypoxic group; HA: hypoxic pseudo-germ-free group.

SUPPLEMENTARY FIGURE S7 Correlation analysis between gut microbiota, fecal metabolites and brain behavioral data. **(A)** Heat map of correlation analysis between latent escape period and gut microbiota in mice (NC vs CA); **(B)** Mouse latent escape period and intestinal metabolites correlation analysis heat map (NC vs CA); **(C)** differential flora and differential metabolites correlation analysis heat map (NC vs CA). (The correlation coefficients *R* are shown in color, where *R* > 0 indicates positive correlation, which is shown in brown or yellow or red, and *R* < 0 indicates negative correlation, which is shown in dark blue or brown or azure; the darker the color, the stronger the correlation). ns, no significant difference; **p* < 0.05 indicates significant difference. NC, control group; CA, control pseudo-germ-free group; HC, hypoxic group; HA, hypoxic pseudo-germ-free group.

SUPPLEMENTARY FIGURE S8 Correlation analysis between gut microbiota, fecal metabolites and brain behavioral data. **(A)** Heat map of correlation analysis between latent escape period and gut microbiota in mice (HC vs HA); **(B)** mouse latent escape period and intestinal metabolites correlation analysis heat map (HC vs HA); **(C)** differential flora and differential metabolites correlation analysis heat map (HC vs HA). (The correlation coefficients *R* are shown in color, where *R* > 0 indicates positive correlation, which is shown in brown or yellow or red, and R<0 indicates negative correlation, which is shown in dark blue or brown or azure; the darker the color, the stronger the correlation). ns, no significant difference; **p* < 0.05 indicates significant difference. NC, control group; CA, control pseudo-germ-free group; HC, hypoxic group; HA, hypoxic pseudo-germ-free group.
