# Supplementary figures and images for: Research on the correlation between gut microbiota and brain cognitive function under chronic hypoxia at high altitude
Source: Front Neurosci. 2025 Jun 19;19:1600069. doi: 10.3389/fnins.2025.1600069 (PMC12222271; doi:10.3389/fnins.2025.1600069)

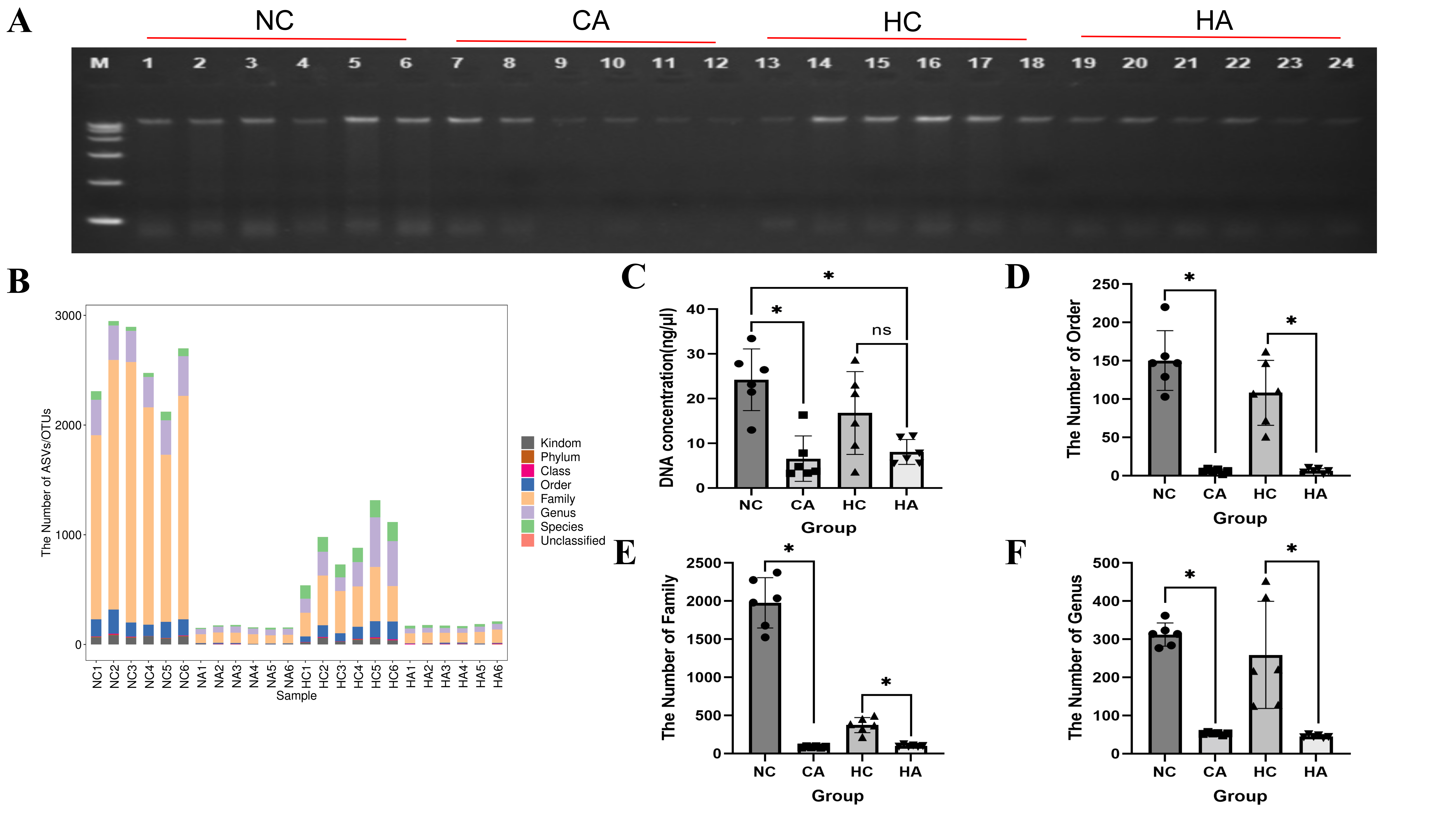

Supplement: SUPPLEMENTARY FIGURE S1 — Quantitative verification of pseudo-germ-free mice. (A) The results of the bacterial DNA gel electrophoresis; (B) The number of ASVs/OTUs in four groups; (C) The results of bacterial DNA content; (D) The number of order in four groups; (E) The number of family in four groups; (F) The number of genus in four groups. ns, no significant difference; *p < 0.05 indicates significant difference. NC, control group; CA, control pseudo-germ-free group; HC, hypoxic group; HA, hypoxic pseudo-germ-free group. [file Image_1.jpeg]

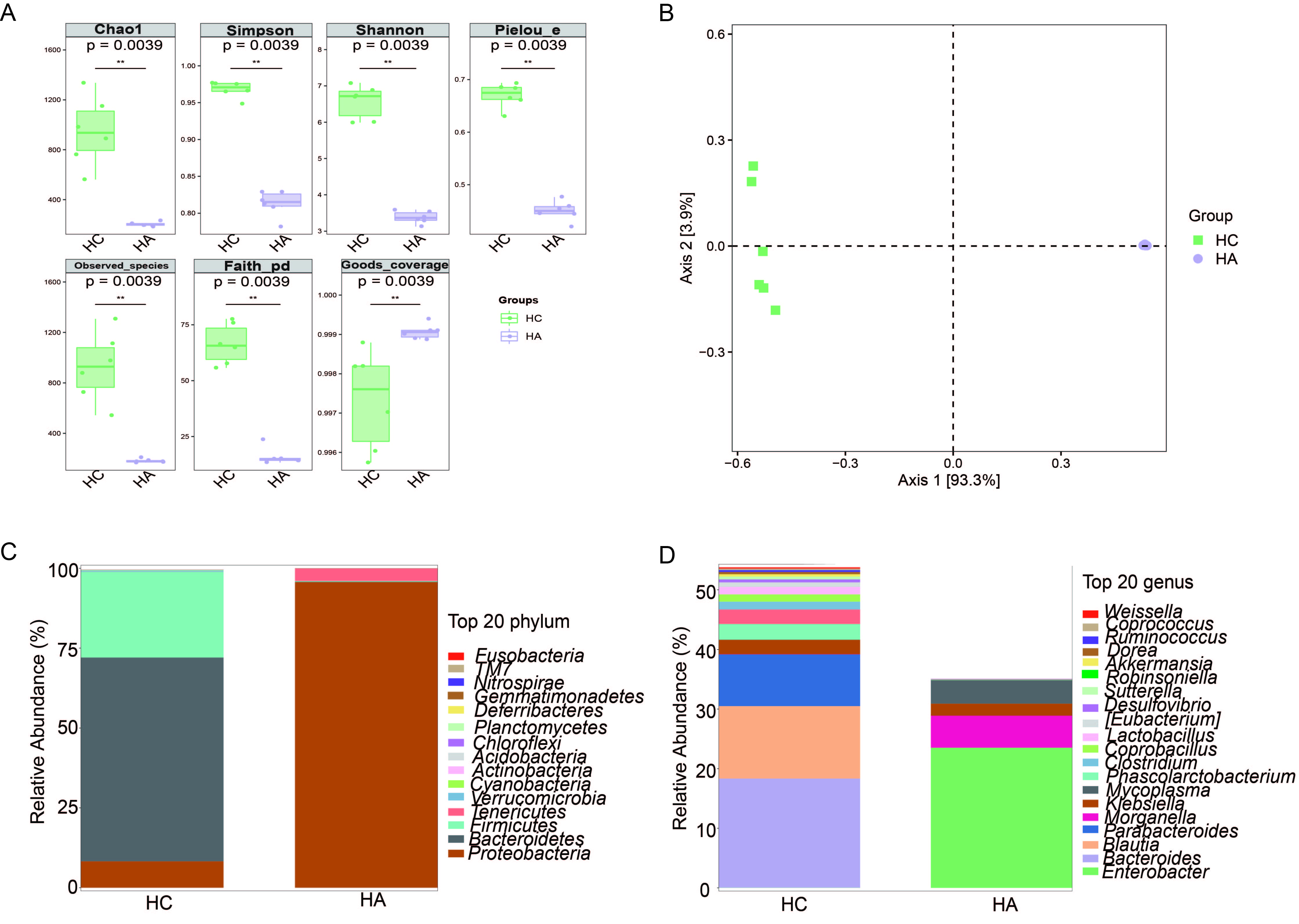

Supplement: SUPPLEMENTARY FIGURE S2 — Changes in gut microbiota of mice. (A) Alpha diversity analysis of gut microbial communities; (B) Principal coordinate analysis (PCoA) of unweighted UniFrac intergroup distances; (C) histogram of abundance at the phylum level of the mouse microbial community. (D) histogram of abundance at the genus level of the mouse microbial community. ns, no significant difference; *p < 0.05 indicates significant difference. NC, control group; CA, control pseudo-germ-free group; HC, hypoxic group; HA, hypoxic pseudo-germ-free group. [file Image_2.jpeg]

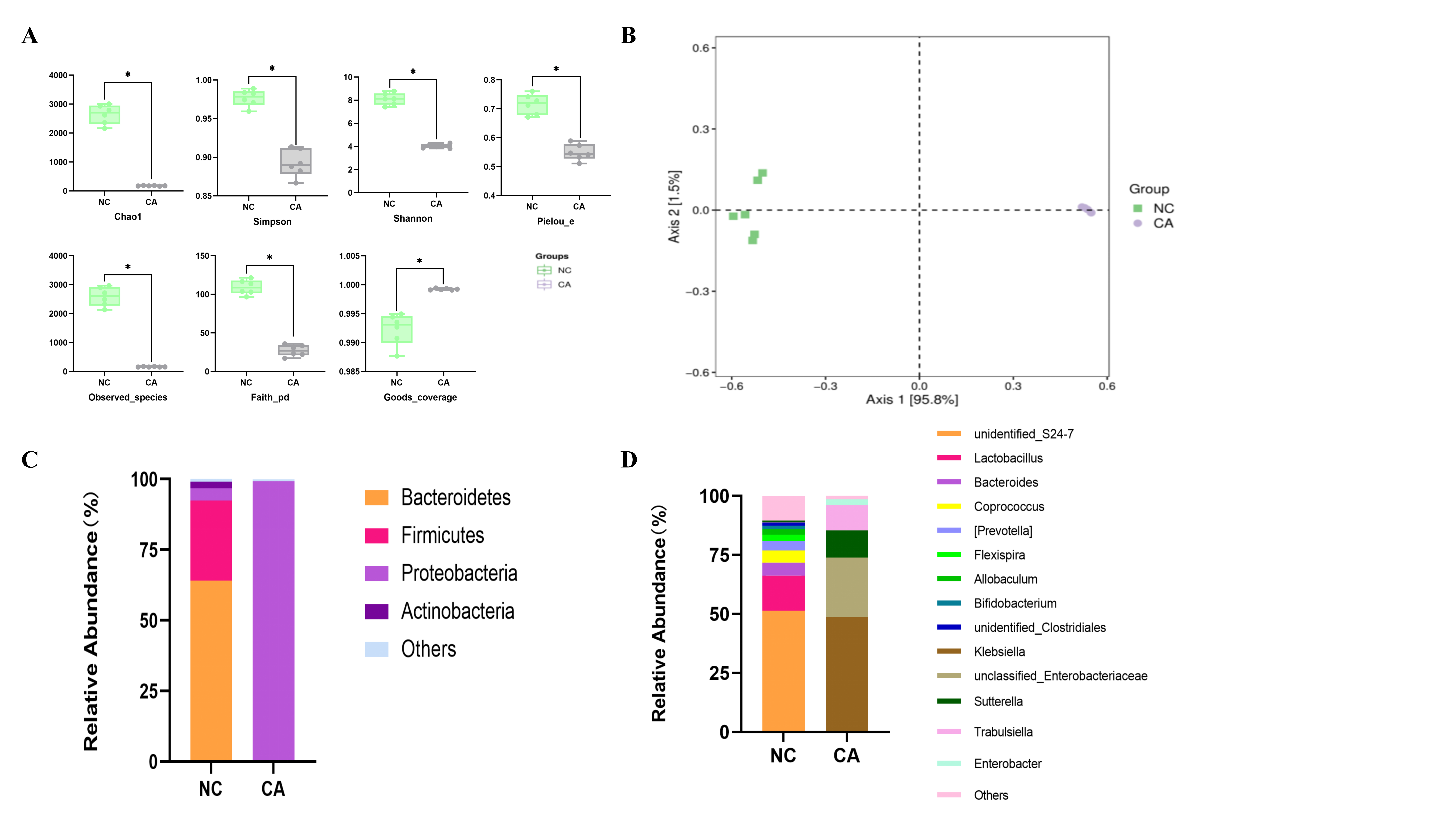

Supplement: SUPPLEMENTARY FIGURE S3 — Changes in gut microbiota of mice. (A) Alpha diversity analysis of gut microbial communities; (B) Principal coordinate analysis (PCoA) of unweighted UniFrac intergroup distances; (C) histogram of abundance at the phylum level of the mouse microbial community; (D) histogram of abundance at the genus level of the mouse microbial community. ns, no significant difference; *p < 0.05 indicates significant difference. NC, control group; CA, control pseudo-germ-free group; HC, hypoxic group; HA, hypoxic pseudo-germ-free group. [file Image_3.jpeg]

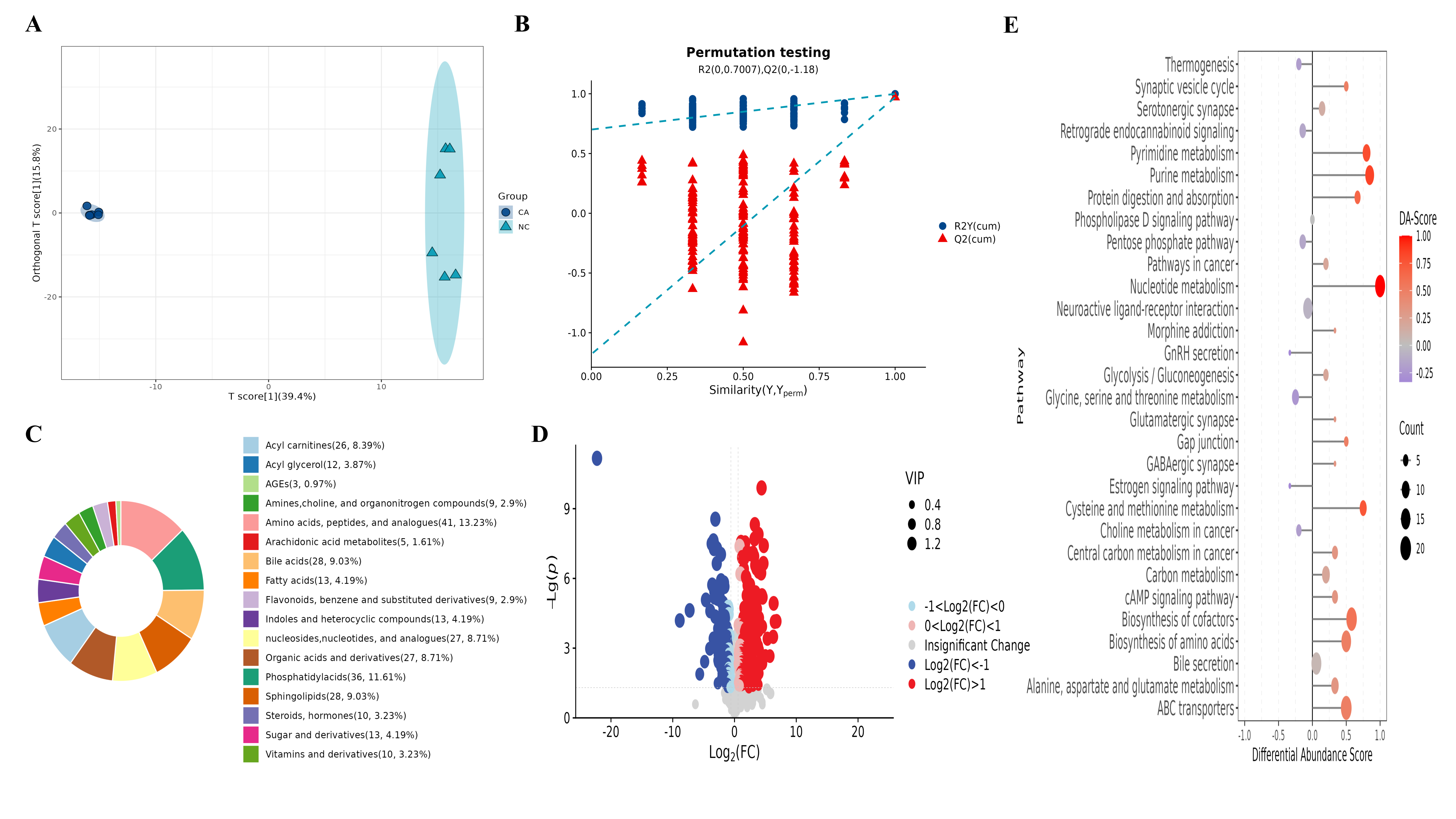

Supplement: SUPPLEMENTARY FIGURE S4 — Changes of metabolites of gut microbiota in mice. (A) Distinction of OPLS-DA model based on the metabolic profiles of gut microbiota (n = 6); (B) Robustness of the OPLS-DA model was assessed by using a randomized permutation test with 200 trials; (C) Differential metabolite classification; (D) Heat maps of the different metabolites in the feces of mice; (E) Differential scoring of KEGG pathway abundance. ns, no significant difference; *p < 0.05 indicates significant difference. NC, control group; CA, control pseudo-germ-free group; HC, hypoxic group; HA, hypoxic pseudo-germ-free group. [file Image_4.jpeg]

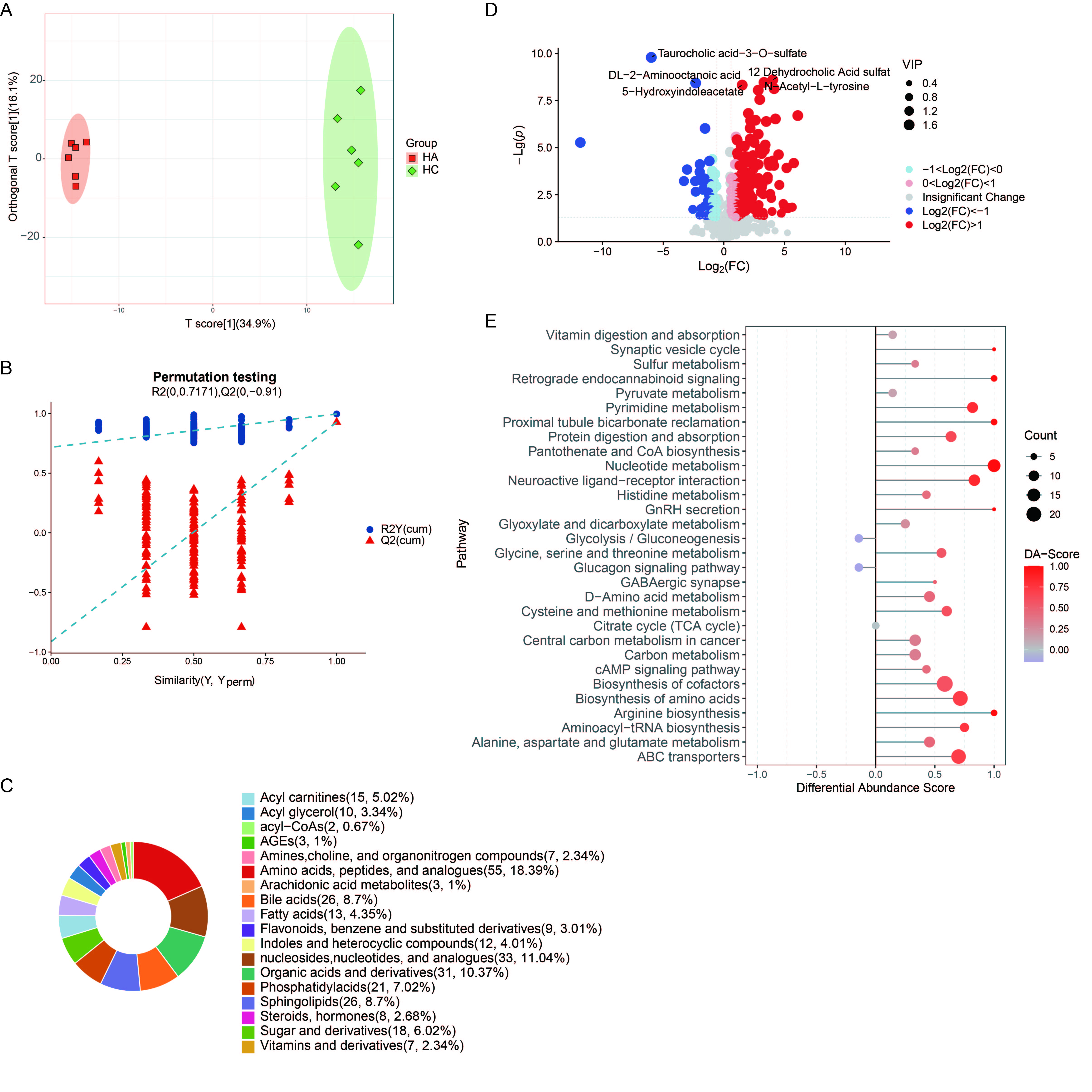

Supplement: SUPPLEMENTARY FIGURE S5 — Changes of metabolites of gut microbiota in mice. (A) Distinction of OPLS-DA model based on the metabolic profiles of gut microbiota (n = 6); (B) Robustness of the OPLS-DA model was assessed by using a randomized permutation test with 200 trials; (C) Differential metabolite classification; (D) Heat maps of the different metabolites in the feces of mice; (E) Differential scoring of KEGG pathway abundance. ns, no significant difference; *p < 0.05 indicates significant difference. NC, control group; CA, control pseudo-germ-free group; HC, hypoxic group; HA, hypoxic pseudo-germ-free group. [file Image_5.jpeg]

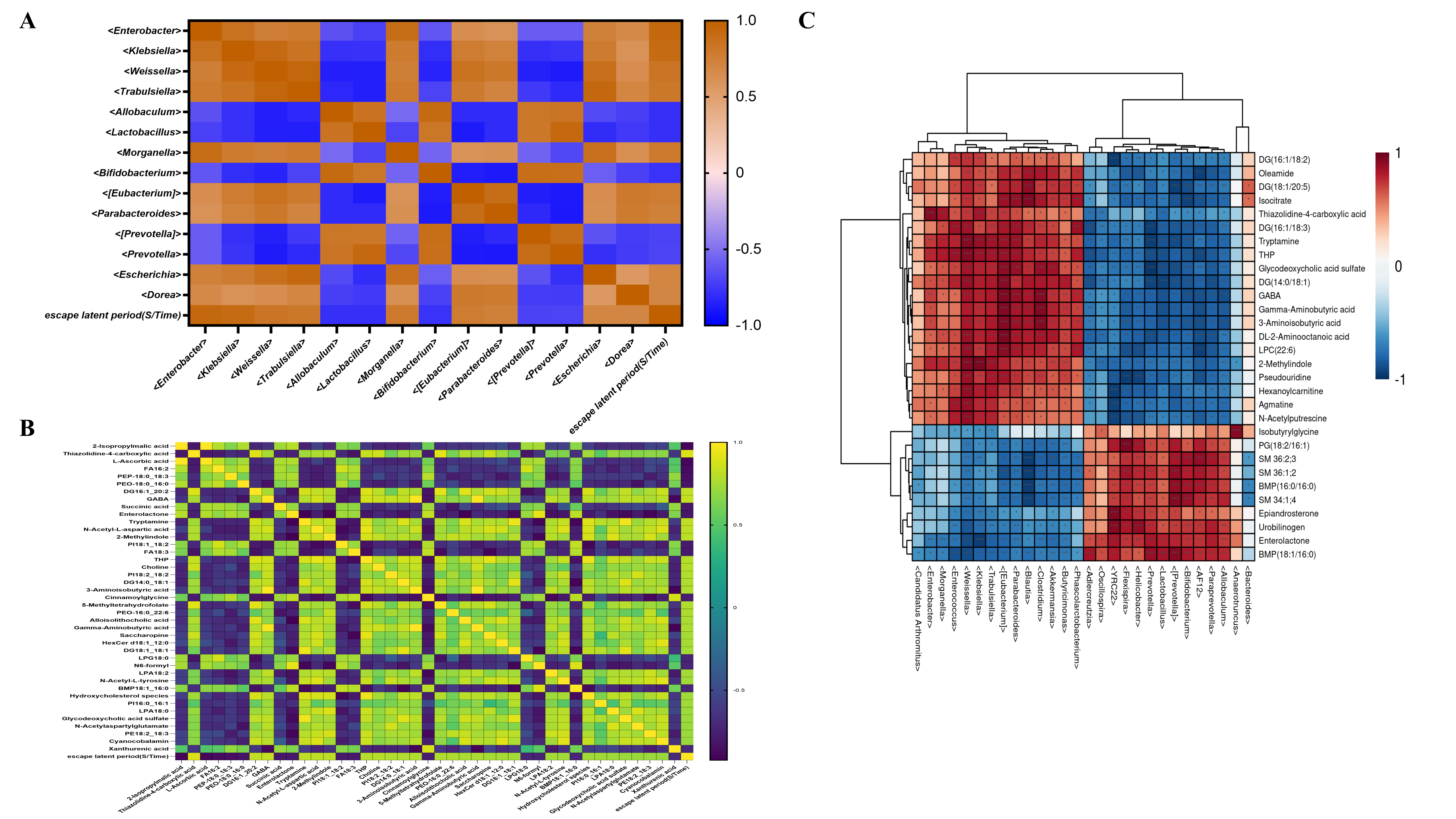

Supplement: SUPPLEMENTARY FIGURE S6 — Correlation analysis between gut microbiota, fecal metabolites and brain behavioral data. (A) Heat map of correlation analysis between latent escape period and gut microbiota in mice (NC vs HC); (B) Heat map of correlation analysis between latent escape period and intestinal metabolites in mice (NC vs HC); (C) differential flora and differential metabolites correlation analysis heat map (NC vs HC). (The correlation coefficients R are shown in color, where R > 0 indicates positive correlation, which is shown in brown or yellow or red, and R < 0 indicates negative correlation, which is shown in dark blue or brown or azure; the darker the color, the stronger the correlation). ns, no significant difference; *p < 0.05 indicates significant difference. NC: control group; CA: control pseudo-germ-free group; HC: hypoxic group; HA: hypoxic pseudo-germ-free group. [file Image_6.jpeg]

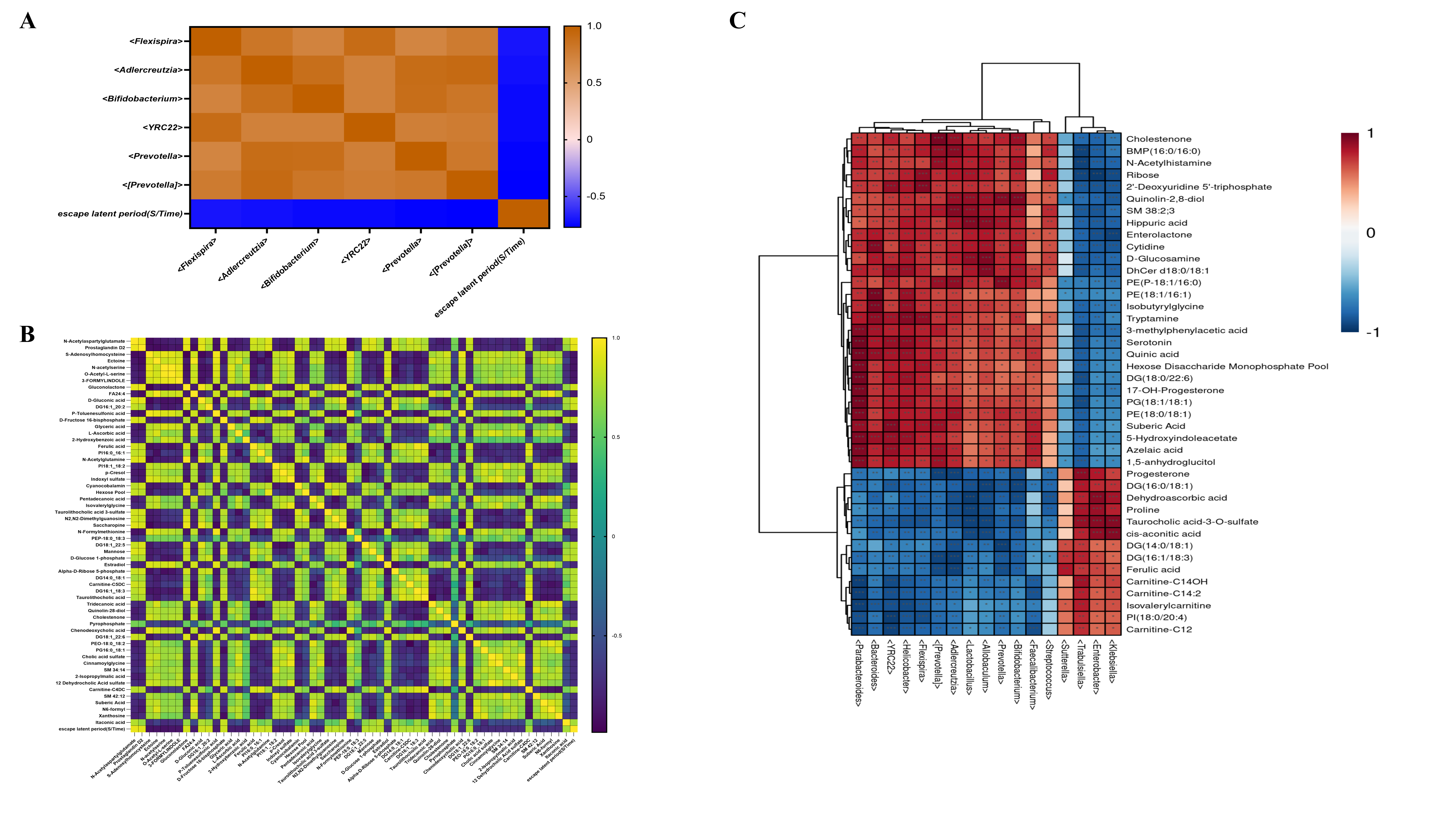

Supplement: SUPPLEMENTARY FIGURE S7 — Correlation analysis between gut microbiota, fecal metabolites and brain behavioral data. (A) Heat map of correlation analysis between latent escape period and gut microbiota in mice (NC vs CA); (B) Mouse latent escape period and intestinal metabolites correlation analysis heat map (NC vs CA); (C) differential flora and differential metabolites correlation analysis heat map (NC vs CA). (The correlation coefficients R are shown in color, where R > 0 indicates positive correlation, which is shown in brown or yellow or red, and R < 0 indicates negative correlation, which is shown in dark blue or brown or azure; the darker the color, the stronger the correlation). ns, no significant difference; *p < 0.05 indicates significant difference. NC, control group; CA, control pseudo-germ-free group; HC, hypoxic group; HA, hypoxic pseudo-germ-free group. [file Image_7.jpeg]

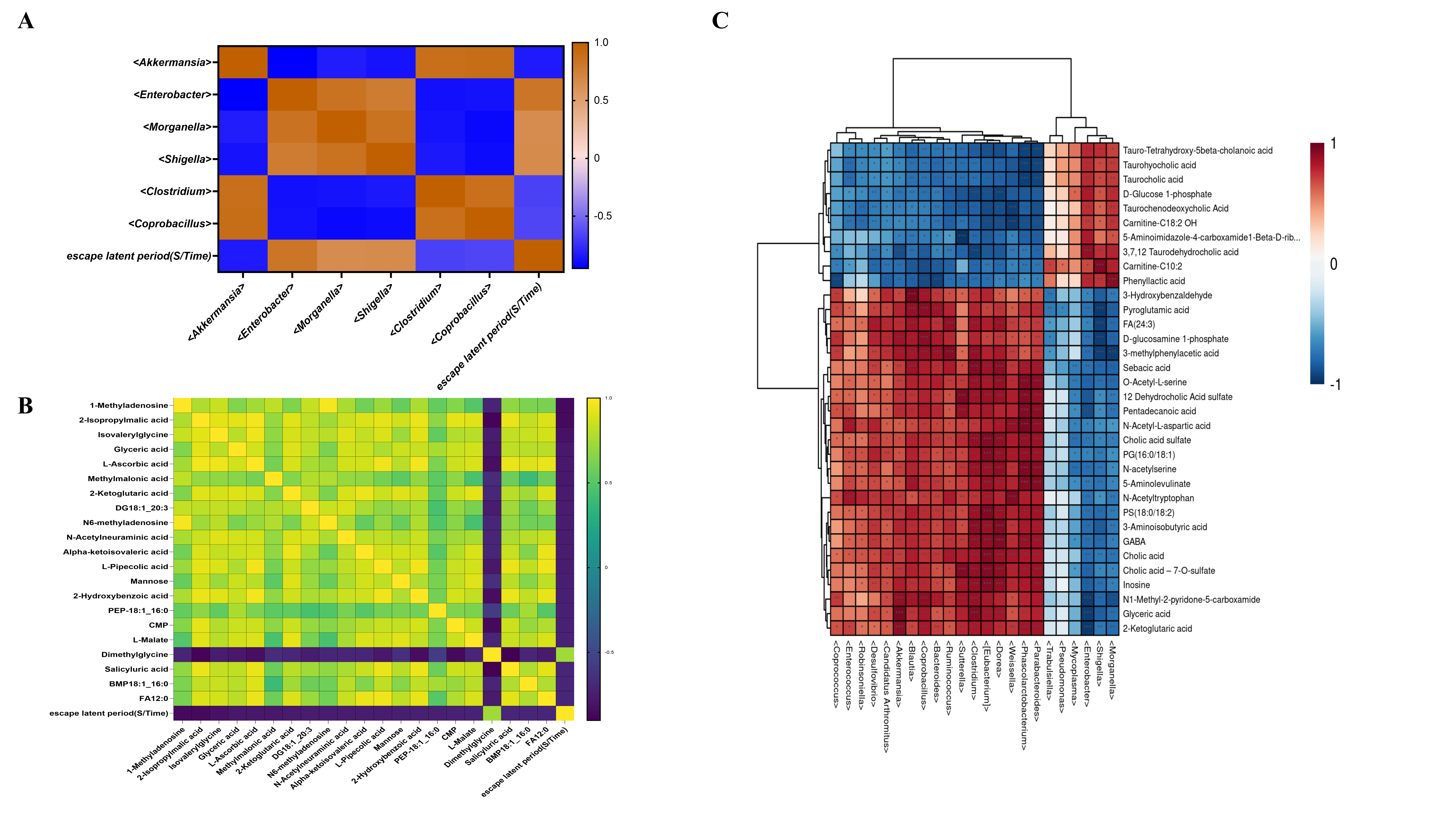

Supplement: SUPPLEMENTARY FIGURE S8 — Correlation analysis between gut microbiota, fecal metabolites and brain behavioral data. (A) Heat map of correlation analysis between latent escape period and gut microbiota in mice (HC vs HA); (B) mouse latent escape period and intestinal metabolites correlation analysis heat map (HC vs HA); (C) differential flora and differential metabolites correlation analysis heat map (HC vs HA). (The correlation coefficients R are shown in color, where R > 0 indicates positive correlation, which is shown in brown or yellow or red, and R<0 indicates negative correlation, which is shown in dark blue or brown or azure; the darker the color, the stronger the correlation). ns, no significant difference; *p < 0.05 indicates significant difference. NC, control group; CA, control pseudo-germ-free group; HC, hypoxic group; HA, hypoxic pseudo-germ-free group. [file Image_8.jpeg]
